# Synthesis and Characterization of BaTiO_3_/Polypyrrole Composites with Exceptional Dielectric Behaviour

**DOI:** 10.3390/polym10111273

**Published:** 2018-11-16

**Authors:** Khalil Ahmed, Farah Kanwal, Shahid M. Ramay, Shahid Atiq, Rabia Rehman, Syed Mansoor Ali, Nasser S. Alzayed

**Affiliations:** 1 Institute of Chemistry, University of the Punjab, Lahore 54590, Pakistan; khalil.icpu@gmail.com (K.A.); grinorganic@yahoo.com (R.R.); 2 Department of Physics and Astronomy, College of Science, King Saud University, P.O. Box 2455, Riyadh 11451, Saudi Arabia; smramay@yahoo.com (S.M.R.); symali@ksu.edu.sa (S.M.A.); nalzayed@ksu.edu.sa (N.S.A.); 3 Centre of Excellence in Solid State Physics, University of the Punjab, Lahore 54590, Pakistan; satiq.cssp@pu.edu.pk

**Keywords:** barium titanate, polypyrrole, dielectric behaviour, polymerization

## Abstract

Higher concentrations of ceramic fillers induce brittleness in the ceramic/polymer hybrids which restrict their applications to limited fields especially when such hybrids are prepared for their use as dielectrics. We have synthesized and characterized different BaTiO_3_-polypyrrole (PPy) composites by changing the concentration of BaTiO_3_ from 1% by weight of PPy taken to 5 wt % to explore its effect on the dielectric parameters of the final product and found that the BaTiO_3_-polypyrrole composite with weight ratio of 0.05:1 exhibited highest dielectric constant, lowest dielectric loss and thermally most stable. All the composites were prepared using in-situ polymerization of pyrrole in an aqueous dispersion of low content of BaTiO_3_ in the presence of small amount of Hydrochloric acid. These composites were characterized for their microstructure and crystallinity by X-ray diffractometer (XRD), Fourier transform infrared (FT-IR) spectroscopy and scanning electron microscopy (SEM) while thermal stability by thermo gravimetric (TGA) analysis. An impedance analyser (LCR meter) was utilized to investigate the dielectric parameters. FT-IR data confirmed the presence of the two phases and their interaction, inferred from the shifting of normal PPy peaks. The data obtained from XRD confirmed the presence of crystallites of 2.8 to 5 nm with dominant crystallinity of the filler, TGA analysis (25 to 600 °C) confirmed the higher thermal stability induced on successive addition of the filler into the prepared composites as compared to that of pure PPy in a wide temperature range which is unusual for such a low % age addition of the filler. The SEM analysis together with XRD results reveal that the successive introduction of BaTiO_3_ particles produced crystallites of 2 to 5 nm size which bonded together and changed the hemispherical shaped larger grains of the matrix to regular shaped smaller grains. The dielectric constant of the composites was enhanced with filler contents from 178 to 522 at 1 MHz for 1 wt % and 5 wt % BaTiO_3_ respectively. It was concluded that the introduction of BaTiO_3_ into the polymer matrix with this new procedure has greatly affected the polymerization process, thermal stability, morphology and dielectric properties of the host matrix and has resulted in a novel series of the composites which may have broad applications.

## 1. Introduction

The rapid development of the electronic industry can, no doubt, be attributed to the success in the synthesis of ceramic/polymer composites with high dielectric permittivity, semi conductivity and electromagnetic interference shielding [1,2,3,4]. These composites must possess low dielectric losses, convenient and low cost processing conditions and excellent thermal stability to utilize these in the devices which can store electrical energy such as capacitors and batteries [5,6]. Besides this a number of other prominent applications of such materials are being exercised in the fields of engineering, biomedicine and military warfare equipment [7,8,9,10,11,12]. The introduction of ferroelectric ceramic particles into different polymers used as host has been reported with their novel electronic properties [13,14,15,16,17,18,19,20]. The ferroelectric particles used to enhance the dielectric properties include BaTiO_3_, TiO_2_, SrTiO_3_, PbTiO_3_and chemical combinations of these [21,22]. The host polymer matrix which have been mostly employed for the purpose of above mentioned requisite properties fall under the category of insulators which have polar nature like PEN (polyarylene ether nitrile), PVDF, PVA, PVC, polyimide and so forth.

Dielectric constant of a material is directly related to its polarizability in the applied field of given strength and is a complex function of a number of variable factors, in addition to the nature of the filler and polymer. Among these, the most important are concentration and particle size of the filler which directly influence the polarization developed at the interfaces. The increase in the dielectric constant is not significant as agglomeration of the particles of the filler at their high loading results in porosity which contain air and the overall dielectric constant decrease from the expected value [23,24]. Higher concentration of the filler also induces brittleness, severe deterioration of mechanical properties and flexibility [25] in the polymer matrix which restricts its applications to a narrow spectrum of electronic devices. The size and morphology of the particles in the final composites, also referred as effective filler, is responsible for the overall behaviour of the composite under consideration and it is not the size of the particles in the powdered filler that was initially introduced [26].

As far as the conducting polymers, such as polyaniline (PANI), polypyrrole (PPy) and so forth, are concerned, their use as the host have been avoided due to the probability of risk of conduction in the applications where short circuiting is the most likely expected. PANI has been filled with magnetic [27,28] and dielectric particles [29] and the composites thus obtained were found to have excellent magnetic and dielectric properties, respectively. Polyptrrole (PPy) being used in gas sensors, photovoltaic cells, conductive fibres and capacitors for its conductivity, physiochemical stability and relatively low cost [30]. In addition to this PPy possess environmental and thermal stability, easy to prepare and exhibits pseudo capacitance [31]. BaTiO_3_ carries the properties like ferroelectricity, Perovskite structure and high dielectric constant [32]. It is also considered lead free, environment friendly and has low manufacturing cost. The combination of these two is likely to meet the requirements of high dielectric constant ceramic polymer composites. Very small amount of polypyrrole (0.3% to 1.4%) has been deposited on the surface of (5.0 g) BaTiO_3_ by Shin Nosuke Miyauchi et al. (1989) using different amounts of FeCl_3_ as oxidant and a dielectric constant of 10^3^ to 10^4^ was reported in the frequency range of 100 Hz–1 MHz. The reason for this much higher permittivity was not clear and it was attributed to the content of polypyrrole [33]. In this regard no investigation into dielectric losses and thermal stability were reported. Similarly in another such deposition of PPy on BaTiO_3_nanoparticles was done by Ipsita Halder and Arabinda Nayak (2017). They prepared 32 wt % PPy-BaTiO_3_ composite and studied its temperature dependent dielectric properties and magnetic properties and concluded that the said composite may be used as magnetic field sensor even at low magnetic field [34]. The study of ferroelectric particles into the conducting polymer matrix has revealed that there is a decrease in conductivity with the increase in the wt % or vol % of the filler [35]. Keeping in view such investigations and focusing the effect of processing conditions on the conductivity of such polymers, the composites using TiO_2_ in PPy and TiO_2_-PVA in PPy [36,37] were successfully prepared and characterized exhibiting excellent dielectric behaviour found both at low and high frequencies of the applied electric field (AC). The trend was attributed to the very small particle size in the final composites which offered large surface to volume ratio for high interfacial polarization.

In the present work, a series of composites have been synthesized and characterized for desired structural, thermal and dielectric properties by introducing only 1 to 5 wt % BaTiO_3_ into the PPy matrix. The novelty of the work lies in the fact that it is not merely the deposition of PPy on BaTiO_3_ but method of synthesis employed was designed to result in the formation of composites which have final crystallite size of less than 5 nm so that such a low concentrations of the filler induce the equivalent dielectric behaviour along with the maintenance of the flexibility of the matrix (PPy) as compared to the previous works where the %ages of the filler were in the range of 5%–50% [33,34]. The dielectric properties were actually focused rather enhancement in conductivity where PPy has always been prepared at 0 °C while for the present work a temperature of 30 °C, acidic and aqueous medium and vigorous stirring under inert atmosphere resulted in formation of high dielectric constant composites with low dielectric losses and high thermal stability. To the best of our knowledge all these conditions have never been combined to prepare PPy composites.

## 2. Materials and Methods

### 2.1. Materials

Pyrrole monomer (Sigma-Aldrich, Bestellen, Germany), distilled every time before use at 130 °C in the absence of sunlight and was kept carefully at 4 °C in dark, barium titanate (Sigma-Aldrich), hydrochloric acid (Merck A.G., Darmstadet, Germany)), ammonium hydroxide (Purity of NH_3_ ≥ 99.99%), ferric chloride hexahydrate (FeCl_3_·6H_2_O) Merck A.G. Double Distilled Water, Ethanol (Merck A.G.).

### 2.2. Synthesis of Polypyrrole (PPy)

0.1 mole of freshly distilled pyrrole was added drop wise into 0.3 M FeCl_3_·6H_2_O solution taken in a three neck flask. The mixture was stirred vigorously under inert atmosphere of N_2_ at 30 ± 1 °C for 1 h. Black coloured precipitates thus obtained were filtered and washed with water, ethanol, 1.0 M NH_3_ solution and finally with water. It was dried at 60 ± 1 °C in vacuum oven for 48 h. 

### 2.3. Synthesis of BaTiO_3_/PPy Composites

BaTiO_3_ (1 wt % to that of pyrrole) was suspended in 1.0 M HCl solution by stirring for 12 h at 30 ± 1 °C. This homogeneous suspension along with 0.3 M FeCl_3_·6H_2_O was stirred for another hour and then transferred to a three neck flask to which 0.1 moles of freshly distilled pyrrole was added drop wise. The similar procedure of reaction conditions, washings, drying and so forth, were adopted to obtain the final composite as mentioned for the synthesis of pure PPy. The composite has been labelled as PPy/1% BaTiO_3_

Similarly four more other composites were prepared using various amounts of BaTiO_3_ as specified in the Table 1.

FTIR Spectra of the prepared samples were taken in normal IR region of 4000–400 cm^−1^ obtained at RT. Samples were dispersed in KBr as a reference in the form of compressed pellets and were analysed using a Burker Vertex 70 spectrometer (Bruker, Yokohama, Japan) X-ray diffraction studies were performed using a Rigaku Ultimate IV X-ray diffractometer (Rigaku, Ohieu, Japan) was used set at scanning speed of 0.02° per minute in the 2θ range of 5° to 80° using CuKα lines. The XRD data was also utilized to estimate the size of the crystallites (D) with the help of well-known equation [38] as given below:

(1) $$D=\frac{k\;\lambda }{\beta \cos\theta }$$

where *k* is the shape factor (≈0.9), λ is the wavelength of the X-ray of Cu Kα radiation (λ = 0.15408) and β is the full width at half maximum of the most intense peak obtained at 2 theta which then gives the value of θ as divided by 2. To study the surface morphology of the composites a scanning electron microscope (SEM, JSM-6610, Jeol, Peabody, MA, USA) was utilized. For dielectric studies an LCR meter (Wayne Kerr, 6500 B, Wayne Kerr, London, UK) was utilized. Compressed pellets were prepared under hydraulic pressure of 12 tons were placed between the electrodes of the capacitor attached to the LCR meter. The meter gives us the values of Capacitance (C) and Resistance (R) at different frequencies (Usually 100 Hz to 10 MHz)

The real part of the dielectric (έ) was calculated from the equation:

(2) $$έ\;=\frac{Ct}{A\varepsilon_{0}}\;$$

where *C* is the capacitance, *t* is the thickness of the sample pellet, *A* is the area of the sample pellet and $\varepsilon_{0}$ is the permittivity of space (8.85 × 10^−12^ F m^−1^).

The tangent of the dielectric loss (tan δ) and the dielectric loss factor ($\varepsilon^{\prime \prime }$) was calculated as:

(3) $$\tan\;\delta \;=\frac{1}{2\pi fRC}\;$$

(4) $$\varepsilon^{\prime \prime }=\varepsilon^{\prime }\tan\;\delta$$

Here *f* is the frequency of the applied A.C., *R* is the resistance and *C* is the capacitance. The real and imaginary parts of the electronic modulus are then calculated applying the formulas

(5) $$M^{\prime }=\frac{\varepsilon^{\prime }}{{\left(\varepsilon^{\prime }\right)}^{2}+{\left(\varepsilon^{\prime \prime }\right)}^{2}}$$

(6) $$M^{\prime }=\frac{\varepsilon^{\prime \prime }}{{\left(\varepsilon^{\prime }\right)}^{2}+{\left(\varepsilon^{\prime \prime }\right)}^{2}}$$

## 3. Results and Discussion

### 3.1. Fourier Transform Infrared (FT-IR) Analysis

**Figure 1:**
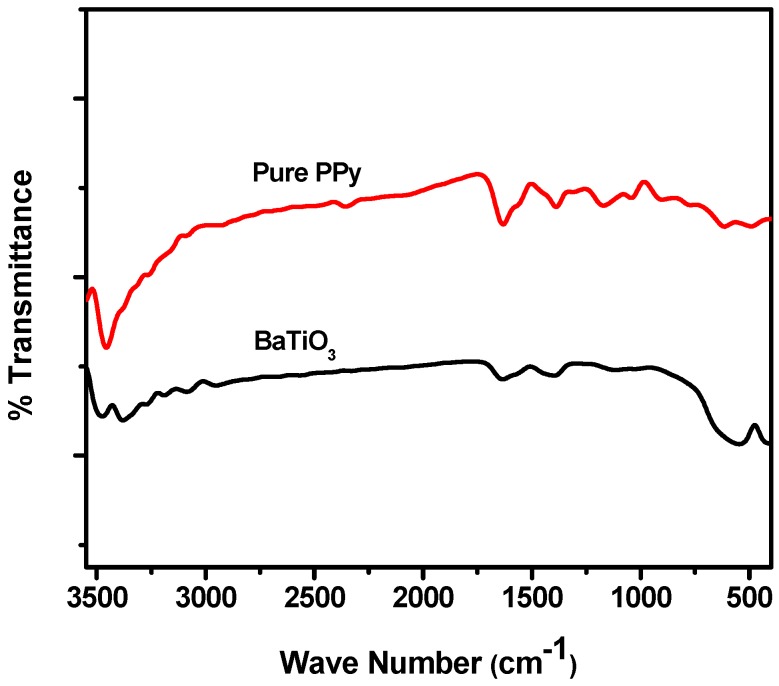
FT-IR spectrum of neat PPy and BaTiO_3._

Figure 1 shows the FT-IR spectra of pure BaTiO_3_ and pure PPy. For the pure BaTiO_3_, two major absorption peaks at 423 cm^−1^ and 550 cm^−1^ correspond to Ti–O bending and stretching vibrations, respectively [39]. The additional absorption peaks of interest are at 1633 and 3473 cm^−1^ may be attributed to moisture absorbed in the concerned material [40]. For the pure PPy prepared the characteristic N–H bond stretch occurs at 3461 cm^−1^ whereas the peaks observed at 1560 cm^−1^ and 1390 cm^−1^ are attributed to C=C and C–N in plane deformation respectively. A medium absorption peak at 1175 cm^−1^ and weak absorption at 1040 cm^−1^ are the responses of =C–C stretching =C–H in plane bending.

**Figure 2:**
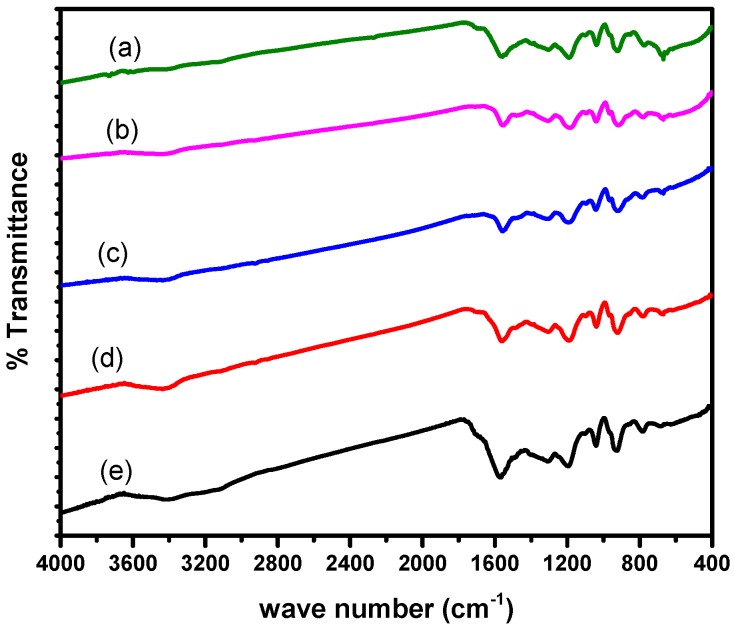
FT-IR spectra of (**a**) PPy/1% BaTiO_3_, (**b**) PPy/2% BaTiO_3_, (**c**) PPy/3% BaTiO_3_, (**d**) PPy/4% BaTiO_3_ and (**e**) PPy/5% BaTiO_3_.

Figure 2 presents the FT-IR spectra of the composites in which at 3415–3430 cm^−1^ a medium band is due to N–H bond stretching of PPy benzenoid rings [41] and this bond seems to have been affected on the introduction of BaTiO_3_ which was observed at 3473 cm^−1^ for pure PPy. Absorption observed at 1560 and 1308–1317 cm^−1^ is the result of stretching due to C=C and in plane deformation of C–N, respectively. The C–N deformation absorption is also in the lower energy region as compared to that of pure PPy (1390 cm^−1^). The absorption peak due to the stretching of =C–C and in plane bending of =C–H is appearing at 1195 and 1040 cm^−1^, respectively. It can be inferred from the shifting of most of the peaks mentioned above for the PPy in the composites towards the shorter frequency that the molar mass of the PPy matrix has increased with associated lengthening of the chain as the wave number or frequency of the absorbed radiations is inversely related to the molar mass according to the well known Hook’s Law. The absorptions due to Ti–O bending vibrations at 423 cm^−1^ and Ti–O stretching vibrations at 550 cm^−1^ attributed to these bonds present in the BaTiO_3_ are observed as weak narrow bands at 446 cm^−1^ and 567 cm^−1^ [42] respectively. All these absorption peaks have been found in connection with the data obtained from the literature concerned [39] and therefore the presence of both phases and *in-situ* formation of the desired composites is confirmed.

### 3.2. X-ray Diffraction Analysis

**Figure 3:**
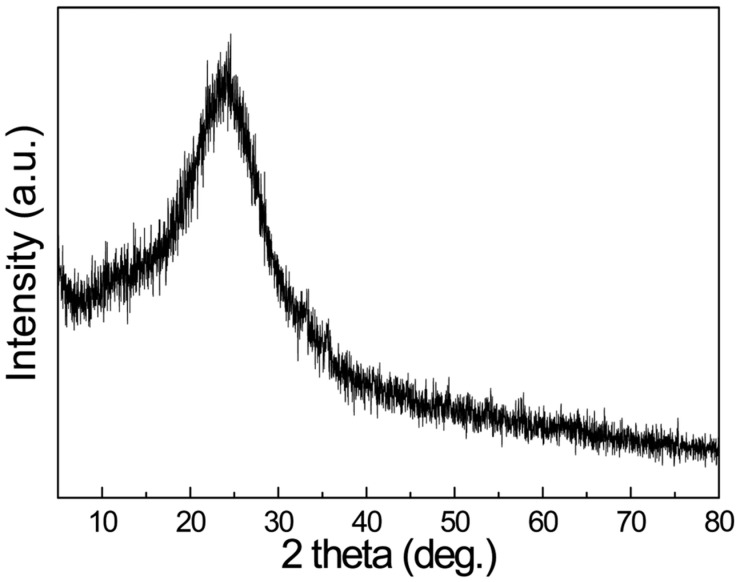
XRD pattern of Pure PPy prepared at 30 °C.

**Figure 4:**
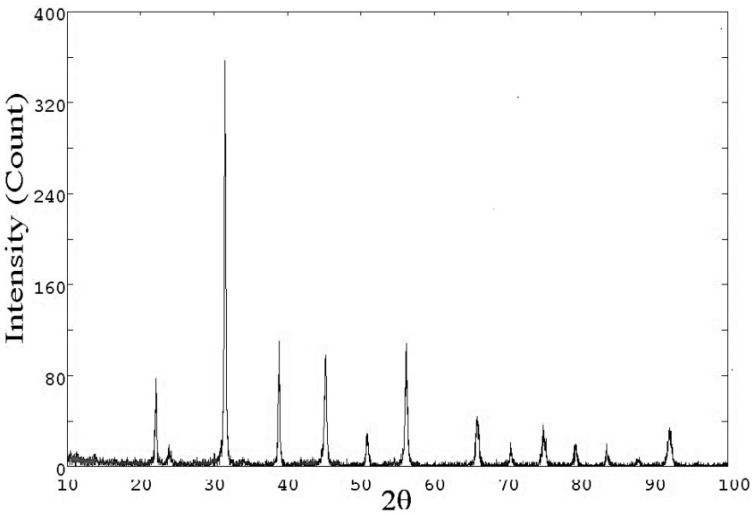
XRD pattern of BaTiO_3_.

**Figure 5:**
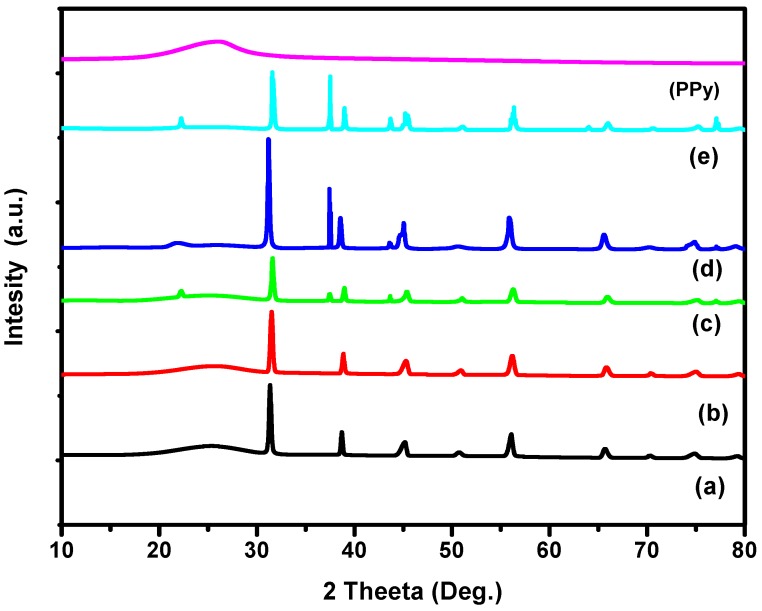
XRD patterns of (**a**) PPy/1% BaTiO_3_, (**b**) PPy/2% BaTiO_3_, (**c**) PPy/3% BaTiO_3_, (**d**) PPy/4% BaTiO_3_ and (**e**) PPy/5% BaTiO_3__._

Figure 3 shows the XRD patterns of pure PPy where reveals its amorphous behaviour and typical diffraction peak at 25.6° [43]. Figure 4 gives XRD pattern of BaTiO_3_ powder employed and Figure 5 of the formulated composites. All the characteristic peaks concerned to the Perovskite tetragonal phase of BaTiO3 are confirmed at 2θ = 22°, 31.4°, 38.8°, 45.2°, 50.9°, 56.2° and 65.8° (JCPDS 05-0626) [44] and (ICSD-29148) [45]. The peaks at 2θ = 22.07°, 31.6°, 38.8°, 45.4° are attributed to the reduction in crystallographic elements in the unit cells of BaTiO3 [44]. The diffraction peaks at 25.4° to 25.7° confirm the presence of PPy being the typical characteristics of the matrix. The pattern is an index to the result that composites are of semi crystalline nature with crystallites of 1 to 5 nm embedded in amorphous PPy matrix [46]. Table 2 shows the values of parameters use d to calculate the crystallite size of the composites and its results.

### 3.3. Thermogravimetric Analysis (TGA)

**Figure 6:**
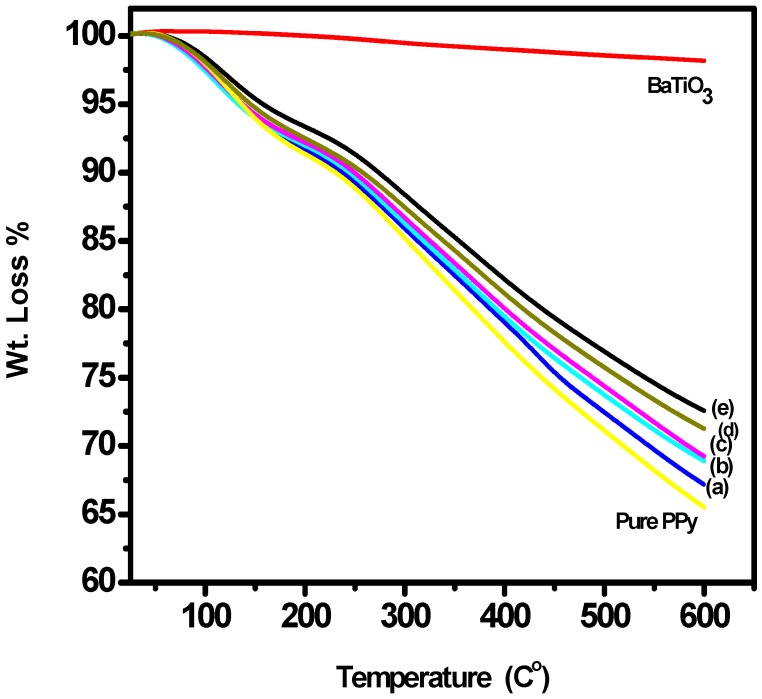
TGA curves of neat PPy, BaTiO_3_ and (**a**) PPy/1% BaTiO_3_, (**b**) PPy/2% BaTiO_3_, (**c**) PPy/3% BaTiO_3_, (**d**) PPy/4% BaTiO_3_and (**e**) PPy/5% BaTiO_3_.

Figure 6 displays TGA curves of pure BaTiO_3_, neat PPy and the five composites prepared. The initial weight loss of about 5% in between 124–133 °C is the result of dehydration of the polymer matrix [47,48]. From the temperature scan of 134–231°C, the weight reduction is because of the beginning of chemical changes thus resulting into the minor decomposition producing the gases like CO_2_ and NH_3_ [49]. In the third stage, the degradation process left still 72% mass in the range 250–600 °C for 5 wt % of BaTiO_3_ (0.335 g in 6.7 g of PPy) which proves its much thermal stability as compared to the pure PPy. Table 3 summarises the wt % age weight losses of the composites with rise in temperature. It is concluded that the introduction of very small amount of BaTiO_3_ induced an overall much higher thermal stability in the composites as compared to those ceramic-polymer composites which have higher wt % ages of the filler [50]. The sharp increase in the thermal stability with the increasing amount of BaTiO_3_ may be attributed to lower mobility of PPy chains when said chains are bound onto the filler particles and chemical interactions developed between PPy and BaTiO_3_ as inferred from FT-IR results.

### 3.4. Scanning Electron Microscopy (SEM) Analysis

**Figure 7:**
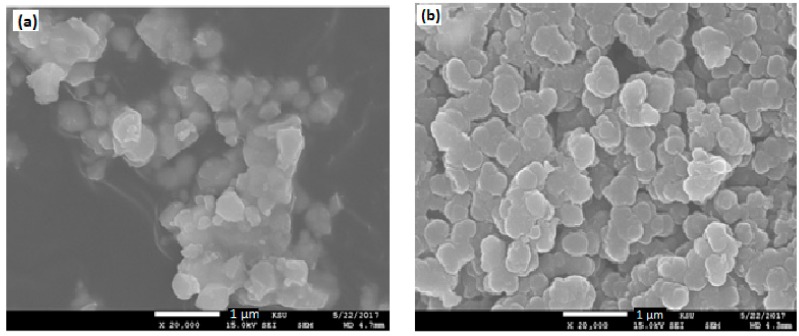
SEM images of (**a**) BaTiO_3_, (**b**) Pure Polypyrrole.

**Figure 8:**
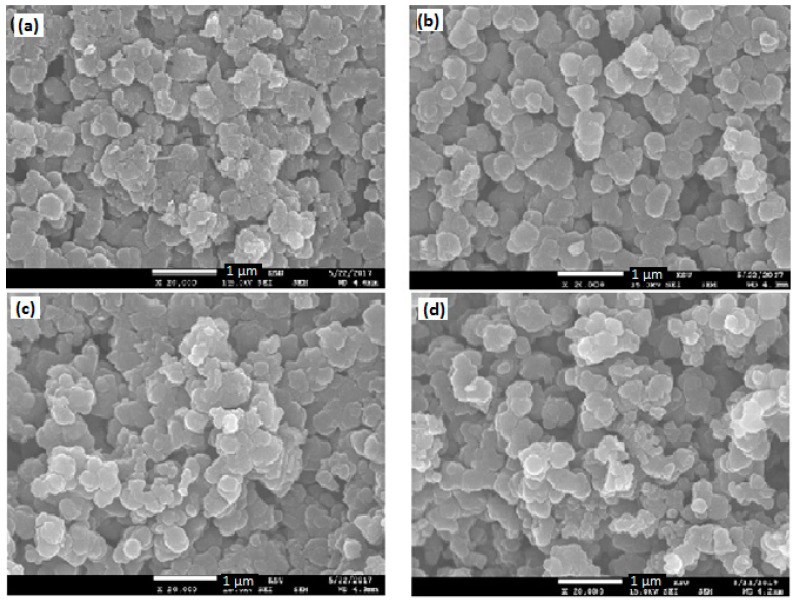

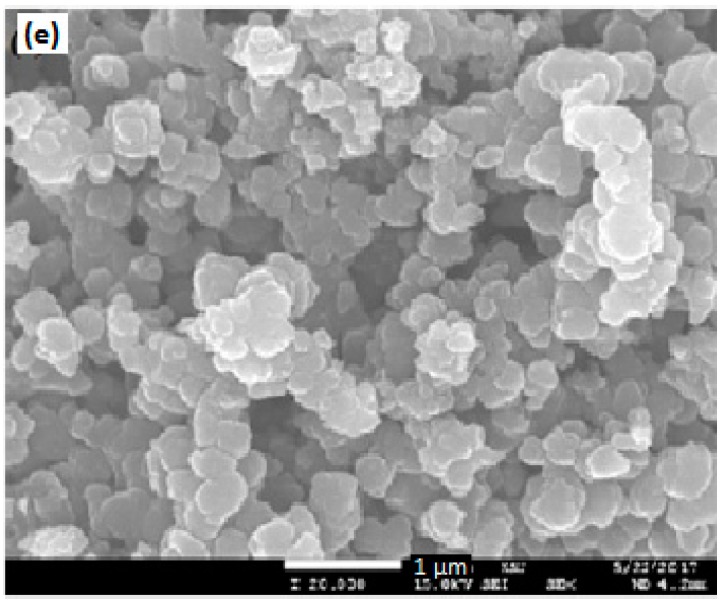
SEM images of (**a**) PPy/1% BaTiO_3_, (**b**) PPy/2% BaTiO_3_, (**c**) PPy/3% BaTiO_3_, (**d**) PPy/4% BaTiO_3_ and (**e**) PPy/5% BaTiO_3_.

Figure 7a,b shows SEM images of Pure BaTiO_3_ and PPy and Figure 8a–e that of PPy/BaTiO_3_ with 1–5 wt % BaTiO_3_ filler, respectively all at the same magnification of 20 k for better comparison of grain size and porosity induced with increase in the amount of the filler. Comparing the Figure 7 and Figure 8 to understand the morphology of the composites, one of the most common features is the aggregation of grains of different sizes and the isolation of the clusters thus produced. In 1 and 2 wt % BaTiO_3_ composites the grains have plate like structure which then immediately changed to granular structure with decrease in grain size, more compactness and an increase in inter granular spaces. When BaTiO_3_ was increased in the range of 3 to 5 wt %. The increase in the filler content has pronounced effect on the bonding between the grains. It is apparent in the SEM image of the final composite having 5% BaTiO_3_ clusters of small granules appear as if these are bridges between different zones. From SEM, FTIR and XRD results we can conclude that the polymerization process has greatly been affected by the introduction of the filler (BaTiO_3_). The pixel ruler estimates the grain size from 0.1 to 0.3 µm and a porosity of 0.5 to 1.0 µm in the composite having 1 wt % BaTiO_3_ (Figure 8a) and a grain size of 0.05 µm to 1.0 µm and interstices of which increased to 1.0 to 1.5 µm in the composite with 5% BaTiO_3_ (Figure 8e). The decrease in grain size and increase in porosity causes increase in interfacial polarization and decrease in the chances of charge transfer from one zone to the other [44,51]. 

### 3.5. Dielectric Studies

**Figure 9:**
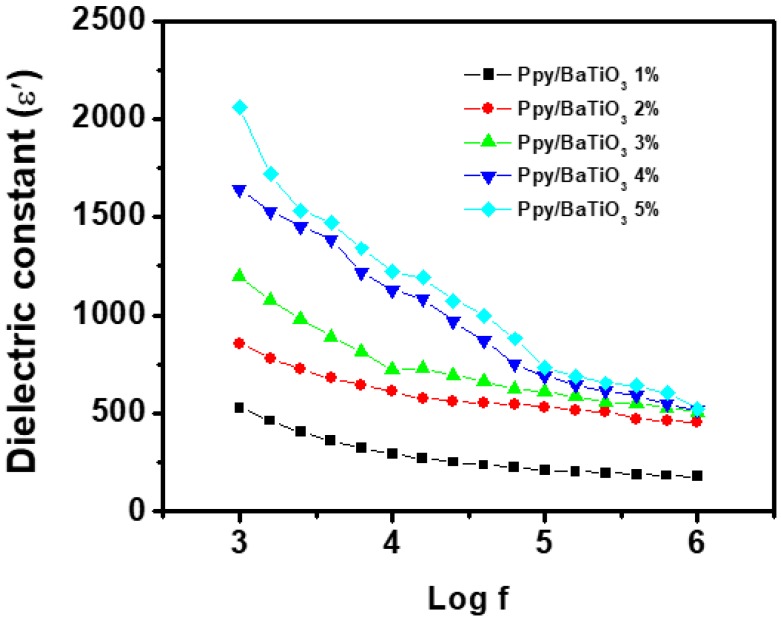
Dielectric constant as a function of AC frequency PPy/BaTiO_3_ composites.

**Figure 10:**
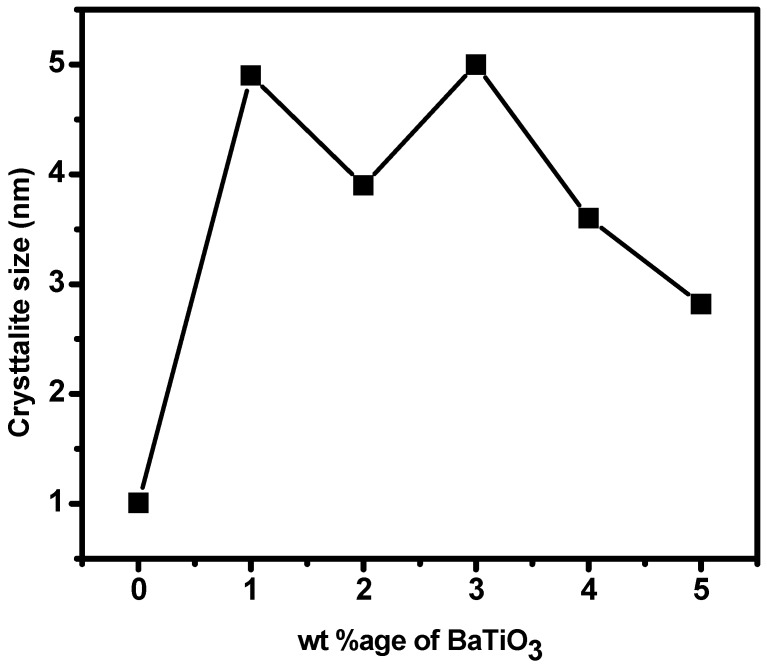
Crystallite size as a function of wt % BaTiO_3_ in the composites.

The dielectric permittivity has always proved as if it was a complicated function of different factors such as type of solids, frequency, temperature and so forth [52]. that is why it varies from material to material. The BaTiO_3_ particles employed were of 2 µm size and the grain size of the matrix as confirmed from the SEM analysis is much lower (0.1 µm to 0.5 µm) still consisting of crystallites of 1 to 5 nm size in the amorphous matrix revealed that the polarization mechanism thus developed has greatly enhanced the dielectric constant at all the frequencies and it continuously increased with the increase in %age by weight of BaTiO_3_. The Figure 9 reveals that the value of dielectric constant was found 529 at 1 kHz and 178 at 1 MHz for 1 wt % BaTiO_3_. The dielectric constant increased to an exceptional value of 2060 at 1 kHz and finally 522 at 1 MHz when the amount of BaTiO_3_ was 5 wt %. An abrupt change in dielectric constant was observed when the amount of the filler was increased to two folds (2 wt %). This may be explained on the basis of the reduction of particle size as evident from Figure 10 where one can see that the particle or crystallite size remained in the range of 4 to 5 nm for composites with 1 wt % to 3 wt % BaTiO_3_ and then reduced to 3.6 nm for 4 wt % BaTiO_3_/PPy and finally 2.82 nm in 5 wt % BaTiO_3_/PPy composite. Smaller particles offer more surface area for polarization phenomena and hence an increase in dielectric constant is observed. Combining the results of crystallite size data obtained from XRD analysis and morphology from SEM images the dielectric constant values are predicted to be high as the charge associated with the individual particles of such a system is localized. The restriction of movement of charges thus produced form an atmosphere of mini capacitors within the composites and then each of these contributes to the overall dielectric constant of the material [51]. 

The FT-IR and XRD datahas also revealed that the composites do not belong to a homogeneous system as the all the characteristic peaks of both components have been obtained. For such a semi heterogeneous system the high dielectric constant values are well explained on the basis of Maxwell-Wagner-Sillars effect [53,54]. When there are two media in contact that possess different conductivities and permittivity space, charges build up at their interfaces [55,56]. Interfacial polarization is mainly a function of intrinsic physical & chemical properties/structure of the filler and intrinsic polarizability of the matrix. In the present work the composites obtained have the filler—matrix strong interfacial interactions which enhanced the dielectric constant [57,58].

**Figure 11:**
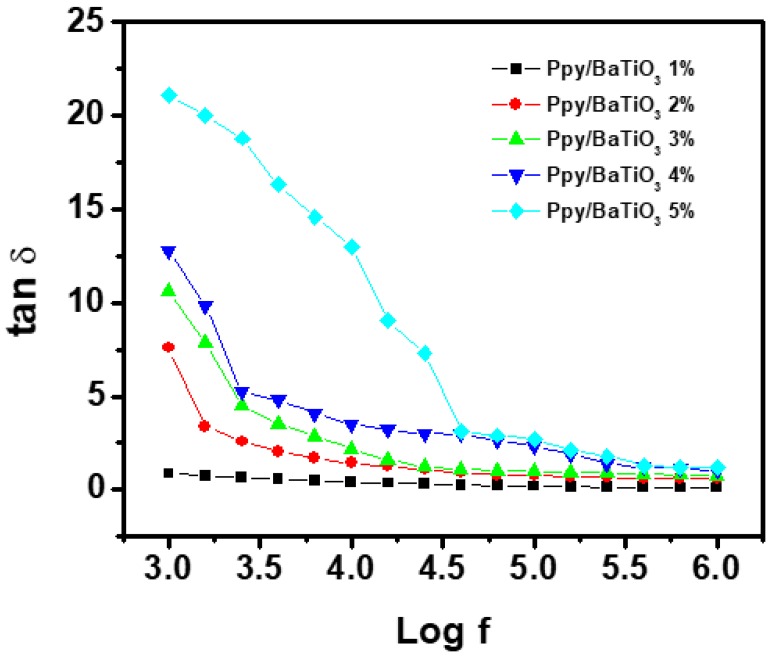
Variation of dissipation factor as a function of AC frequency for PPy/BaTiO_3_ composites.

**Figure 12:**
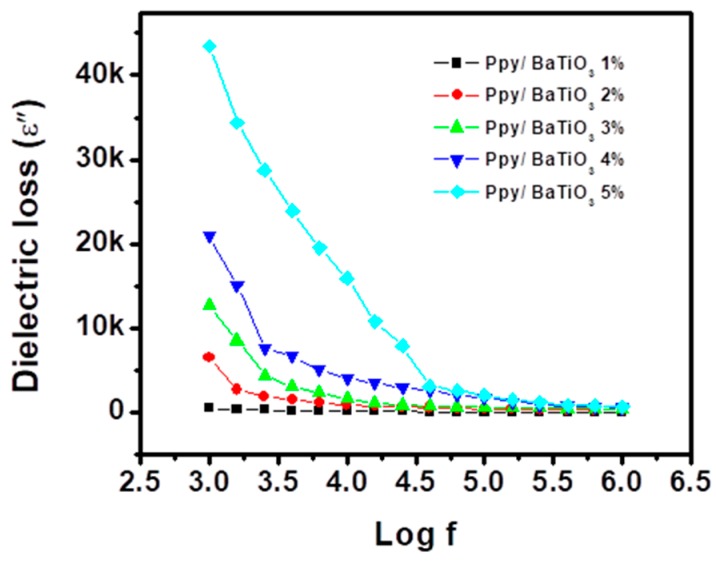
Variation of dielectric loss with frequency for PPy/BaTiO_3_ composites.

Figure 11 shows variation in dissipation factor (tan δ) and Figure 12 depicts dielectric loss which is defined as the combined parameter of the dielectric constant and dissipation factor (εʺ) with frequency and amount of the filler. It can be seen that at 1 kHz, the value of tan δ was 20 times greater for 5% BaTiO_3_ composite as compared to 1 wt % BaTiO_3_ composite which then reduced to 3 times at 1 MHz. The final value of tan δ for the composite with the highest obtained dielectric constant is 1.2 which indicates that the composite has very low heat/dissipation factor which is almost always desired.

The polarization at the interfaces of the amorphous and crystallite phases and the molecular polarity especially due to N–H bond is the major cause of dielectric losses [54]. As shown in the SEM micrographs the porosity has greatly increased in the 5% of BaTiO_3_/Ppy which restricted the charge transport between the grains, the major cause of dielectric loss (εʺ) [51].

These dielectric parameters can well be explained if the composite media are considered under Koop’s theory and Maxwell-Wagner model in which the polarization is almost always developed by the electrons roaming from the grains to their boundaries/edges under the electric field. The increase in frequency causes an increase in the number of hurdles for the electrons to reach the grain boundaries, thus decreasing the polarization and the subsequent fall in dielectric constant.

**Figure 13:**
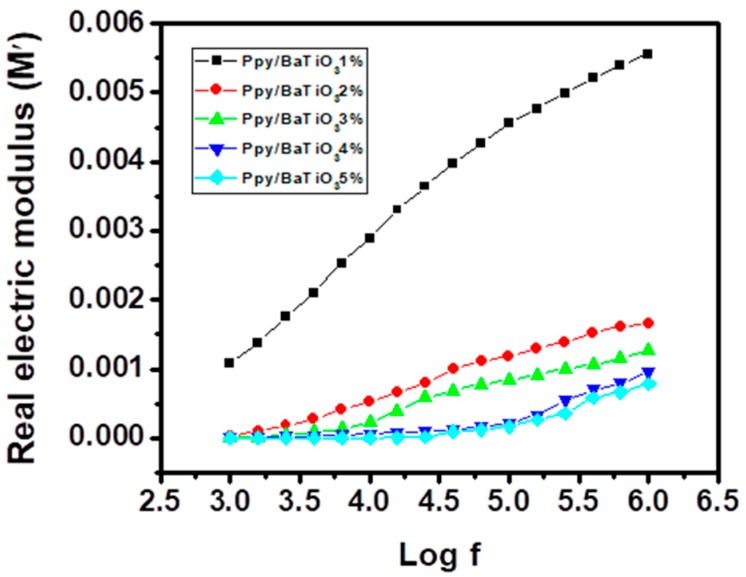
Variation of real part of electric modulus with frequency for PPy/BaTiO_3_ composites.

**Figure 14:**
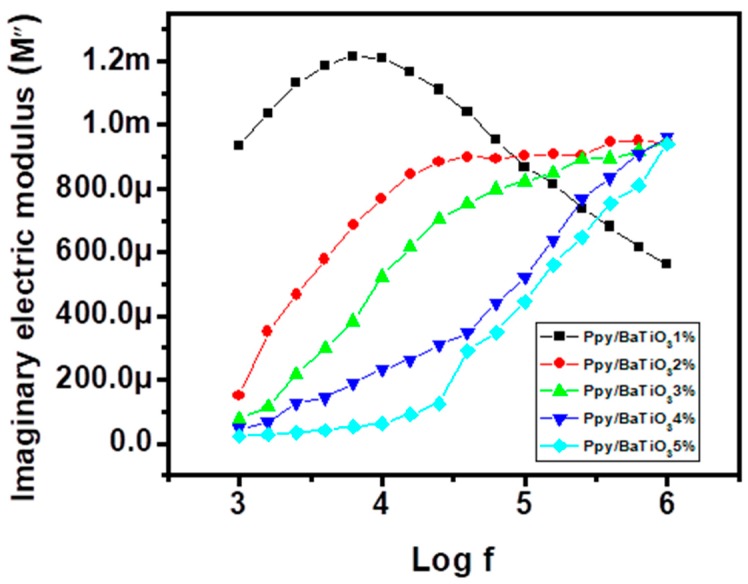
Variation of imaginary part of electric modulus with frequency for PPy/BaTiO_3_ composites.

Figure 13 and Figure 14 presents the frequency dependence of the real and imaginary parts of the dielectric modulus of BaTiO_3_/PPy composites. These parameters enable us to interpret the interfacial polarization and chain segmental relaxation phenomena under the influence of varying electrical field [59,60]. The relaxation time distribution increases after 100 kHz and are maximum at 1 MHz as far as the measurements are concerned. The peak maximum for the imaginary part of the dielectric modulus (M′′ is visible only for 1 wt % BaTiO_3_/Ppy composite and for the other composites (2 to 5 wt % BaTiO_3_/Ppy) cannot be interpreted as the interval of the frequency was not wide enough to present these. This type of behaviour of a system at higher frequency reflects its higher conductivity under the said circumstances and can be attributed to huge number of nanoscale PPy particles which induces semi conductivity [61]. The shift of these peaks maxima towards higher frequencies with increase in the filler is a sign of decrease in molar mas, poor relaxation and in turn the flexibility of the composites [62,63].

When the results of the dielectric behaviour of the composites of BaTiO_3_ and PPy are compared with those of the previously published similar PPy composites [33,34,64,65,66,67] we find that such results have been obtained either through the higher weight or volume percentages of the filler into PPy or by the addition of a third component. These methods induce brittleness and high dielectric losses and make the processing conditions expensive and difficult. In addition to this their application was found almost limited to the capacitors and electromagnetic shielding (EMS)

## 4. Conclusions

High dielectric constant composites of BaTiO_3_-PPy with low dissipation factor and high thermal stability of were synthesized using aqueous dispersion of small amounts of the filler via in situ polymerization. The FT-IR confirmed the formation of PPy in all the composites with characteristic absorption peaks of BaTiO_3_ and it was inferred that the filler particles were partially wrapped by the polymer matrix. The same conclusion was drawn from the XRD studies which also revealed that there are present both amorphous and crystallite phases. With the help of Scherer’s equation, the average sizes of the crystallites in the composites formulated were also calculated and were found in the range of 1–5 nm, which should be the major reason for the dielectric behaviour of the synthesized products, as such particles can serve as mini capacitors within the composite. TGA revealed that the thermal stability of PPy increased with a small amount of BaTiO_3_, which, to the best of our knowledge, is comparable to the composites reported earlier with very high percentages of the ceramic fillers. Dielectric parameters were found exceptional and a value of dielectric constant of 522 and dielectric loss of 1.2 were achieved for the PPy/5% BaTiO_3_ composite (with weight ratio of BaTiO_3_:PPy as 0.05:1) at 1 MHz frequency where the values are usually mentioned in the literature. These parameters along with electric modulus analysis (M′ and M′′) when related to particle size and morphology can account for the electrical properties. It was confirmed that the Maxwell-Wagner-Sillars (MWS) polarization existed in the systems which has been described in the section on dielectric properties. It is also concluded that the composites are suitable for use at both low and high frequencies as dielectric medium under AC electric field.

## Figures and Tables

**Table 1:** Amounts of BaTiO_3_ and PPy in the composites 2–5.

Composite No.	Composite Label	Weight of BaTiO_3_ (mg)	Weight of PPy (mg)	Weight Ratio of BaTiO_3_:PPy
1	PPy/1% BaTiO_3_	67	6700	0.01:1
2	PPy/2% BaTiO_3_	134	6700	0.02:1
3	PPy/3% BaTiO_3_	200	6700	0.03:1
4	PPy/4% BaTiO_3_	268	6700	0.04:1
5	PPy/5% BaTiO_3_	335	6700	0.05:1

**Table 2:** Estimated crystallite sizes for PPy/BaTiO_3_ (1%–5%) composites.

Sample	2θ Value of Max. Intensity Peak	FWHM	Crystallite Size
PPy	26.12°	8.02°	1.01 nm
PPy/BaTiO_3_ (1%)	31.3°	1.68°	4.9 nm
PPy/BaTiO_3_ (2%)	31.5°	2.02°	3.9 nm
PPy/BaTiO_3_ (3%)	31.6°	1.65°	5.0 nm
PPy/BaTiO_3_ (4%)	31.2°	2.29°	3.6 nm
PPy/BaTiO_3_ (5%)	30.6°	2.92°	2.82 nm

**Table 3:** Weight loss data from TGA analysis of PPy/BaTiO_3_ composites at various temperatures.

Temp (°C)	Weight Loss (%) of PPy/BaTiO_3_ Composites					
	PPy	PPy/BaTiO_3_-1% (0.067 g)	PPy/BaTiO_3_-2% (0.134 g)	PPy/BaTiO_3_-3% (0.20 g)	PPy/BaTiO_3_-4% (0.268 g)	PPy/BaTiO_3_-5% (0.335 g)
200	9.8	9.5	9.2	8.8	8.6	8.3
300	15.0	14.9	13.8	13.4	12.6	11.7
500	28.9	22.4	20	19.6	19	18.9
550	31.8	28.9	27.6	26.3	25.7	24.3
595	34.3	34.6	30.6	28.9	28.6	27.3
